# Editorial Notes

**Published:** 1901-03

**Authors:** 


### Editorial Notes.

**A West of England University in Bristol.**—We sincerely hope that ere the new century has grown much older, the South and West of England will be duly represented by a University in Bristol, for if this part of the United Kingdom is to keep abreast of the times and in line with the rest of England, Scotland, Ireland, and Wales, such a University must come within the range of practical politics. Doubtless the older Universities will long maintain their well-established lead as the seats of higher classical and theological scholarship, if not also that foremost position in science towards which they are advancing; but it is now generally recognised that local Universities are equally essential for the promotion of higher and technical education throughout the Kingdom. We are constantly reminded that there is room—and, indeed, ample material—for such a local University in Bristol. Thus in our last issue we had occasion to report some very pertinent remarks on this question which were uttered by Sir William Broadbent in his speech at the University College Medical School dinner. More recently the speeches at the University College Colston Society dinner, and especially that of Sir Michael Foster, quoted below, have brought this important topic into prominent notice.

The second annual dinner of the **University College Colston Society** was held on January 15th, in the New Lecture Hall. The principal guests of the evening were Sir Michael Foster, M.P., and Sir William Church, Bart., M.D. There was a large attendance, and the funds of the College received a considerable augmentation. Sir Michael Foster said he regarded Universities as the most important things in the world. They were flooded with schools, and of all their schools he ventured to assert that the Universities were the heart or the head. Learning acted on the principle which was very well known in another matter as downward filtration. Unless they had their Universities right, it was no good trying to get their schools, of whatever grade, right. Therefore he repeated that it was necessary for the welfare of the nation that its Universities should not only flourish, but should abound. He understood that at Bristol they did not possess a University, but a University College. The great difference between a University and a University College was that the former had the power to confer degrees. One idea in the establishment of Universities in the renaissance was that each University was a guild for the advancement of learning, and that had, with various changes, remained their object ever since. They were institutions for the advancement of learning—not only for the spread of knowledge, but for the pushing forward of the known into the unknown. They had been through all time, with little variation, the great instrument of inquiry and research. A teaching body, whether it was called a College or a University, was a University in principle and in reality if the whole of its actions were governed by the dominant principle of research, of inquiry into the unknown. If every member of their teaching staff felt it was his duty, not merely to spread knowledge, but to increase knowledge, then their institution would not be a mere mechanical institution, but what he might call a spiritual organisation as well. Every University ought to try to be something more than a mere machine for converting knowledge into pap, to be deposited into the open mouth of the student He would venture to suggest to the citizens of Bristol that they had in their midst a body which was practically carrying on University teaching — teaching which he knew from personal knowledge was truly inspired with that feeling upon which he had dwelt—and that such an institution, which must have a great influence upon the intellectual progress and industrial advancement of the West of England, ought to be founded not upon the shifting sands of annual subscriptions, but upon the solid rock of permanent endowment. He also added that the metabolism of these Universities was rapid, and that the need for an abundance of pabulum was great.

The subject of "**A University for Bristol**" was the one chosen for discussion at the meeting of the Bristol Medical Debating Society on December 5th, 1900, when Mr. Richardson Cross gave an address, the object of which was to show that it is essential that the educational opportunities for Bristol and the counties in the West of England should be put on a level with the North of England and the Midlands, and that a University should be established so soon as we can procure the necessary sum of money, and show that our opportunities for teaching are equal to any standard that could be fairly imposed. Birmingham has set us an excellent example. It will be easy to follow when the generous donor has provided the necessary two or three hundred thousand pounds.

At the annual meeting of the Governors of the **University College, Bristol**, held on November 21st, 1900, the Lord

Bishop of Hereford joined in the feeling of satisfaction with which speakers had looked forward to possible united action between the authorities of the Merchant Venturers' College and the University College, who, in friendly conference, endeavoured to make out a working plan which would bring the two colleges into close and friendly co-operation. His belief was that these men were doing one of the greatest and best educational services done in their time for the city of Bristol. Such cordial co-operation between the University and the pioneer technical school of the Merchant Venturers would go far towards smoothing the way for the initiation of a University scheme, for the work at both these institutions is worthy of University status.

**Sanatorium for Consumptives.**—The local branch of the National Association for the Prevention of Consumption and other forms of tuberculosis has been making progress of late. Arrangements had been made for the holding of meetings in various parts of the three counties—Gloucester, Somerset, and Wilts; some of these meetings had been held, but others had been postponed in consequence of the death of the Queen. One of the most influential of these meetings was that which took place at Salisbury on December 13th, 1900, under the presidency of the Lord Bishop of Salisbury, when Sir William Broadbent, Bart., M.D., gave an address. Dr. Shingleton Smith and Dr. Lionel Weatherly also spoke. Other meetings are to be arranged shortly, and meanwhile the Executive Committee of the three counties branch has resolved that steps should be at once taken to establish a sanatorium for consumptives of the poorer classes at a spot which will be in a central position for the three counties.

The site selected is at Winsley, on high ground opposite Limpley Stoke, and not far from Bradford-on-Avon. The site, which comprises fifty acres of land admirably adapted for the purpose, can be obtained for £2,000.

The cost of building and equipment is estimated at £15,000, and the annual cost of maintenance will be about £4,000. The Committee is asking for the support of all who are interested in this important matter, and hope that some generous donors will be found who will speedily complete the object in view.

On January 9th the members of the **Bristol Medico-Chirurgical Society** enjoyed the great advantage of hearing Dr. Patrick Manson, C.M.G., F.R.S., F.R.C.P., give an address on the Life History of the Malarial Parasite. The lucidity of the lecture, and the excellence of the microscopical preparations and lantern slides which were shown, left nothing to be desired. Dr. Manson was modestly silent with regard to his own work in this field of research, but we believe that it was Dr. Manson who first promulgated the view that the mosquito was the intermediary host of the malarial parasite. Subsequently Major Ross, working upon the lines laid down by Dr. Manson, proved that the views which had been expressed by the latter were in a large degree correct.

**The New Sydenham Society.**—The Council proposes to publish an Atlas of Clinical Medicine, Surgery and Pathology. From estimates of cost which have been prepared it is believed that with a membership-roll of two thousand, five fasciculi of this Atlas, of eight plates each, could be afforded annually, and in addition, at least one printed volume. At present the Society has only 1,200 members, and unless an addition of 800 can be made the proposal cannot be carried out. Mr. Jonathan Hutchinson, 15 Cavendish Square, W., will be glad to receive the names of new subscribers.

